# Prehospital assessment and management of postpartum haemorrhage- healthcare personnel’s experiences and perspectives

**DOI:** 10.1186/s12873-021-00490-8

**Published:** 2021-08-28

**Authors:** Ann-Chatrin Linqvist Leonardsen, Ann Karin Helgesen, Linn Ulvøy, Vigdis Abrahamsen Grøndahl

**Affiliations:** 1 Department of Health and Welfare/Ostfold Hospital Trust, Ostfold University College, Postal box code (PB) 700, NO-1757 Halden, Norway; 2 Ostfold Hospital Trust, Department of Anesthesia, 300 NO-1714 Grålum, PB Norway; 3 Ostfold Hospital Trust, Prehospital Department, 300 NO-1714 Grålum, PB Norway

**Keywords:** Postpartum hemorrhage, Manual aortic compression, Prehospital, Ambulance, Questionnaire, Delphi technique, Self-efficacy, Collective efficacy

## Abstract

**Background:**

Postpartum hemorrhage (PPH) is a serious obstetric emergency, and one of the top five causes of maternal mortality globally. The most common causes of PPH include uterine atony, placental disorders, birth trauma and coagulation defects. Timely diagnosis and early management are critical to reduce morbidity, the need for blood transfusion or even mortality. External, manual aortic compression (AC) has been suggested as an intervention that reduce PPH and extend time for control of bleeding or resuscitation. This procedure is not commonly utilized by healthcare personnel. The incidence of home-births is increasing, and competence in PPH assessment and management is essential in prehospital personnel. The objective was to explore prehospital personnel’s competence in PPH and AC, utilizing different tools.

**Methods:**

The study was conducted in a county in South-eastern Norway, including five ambulance stations. All prehospital personnel (*n* = 250) were invited to participate in a questionnaire study. The questionnaire included the PPH self-efficacy (PPHSE) and PPH collective efficacy (PPHCE) tools, as well as tool developed utilizing the Delphi technique. Descriptive statistics were used to analyze the quantitative data, while quantitative content analysis was used to analyse free-text responses.

**Results:**

A total of 87 prehospital personnel responded to the questionnaire, 57.5% male, mean age 37.9 years. In total, 80.4% were ambulance workers and/or paramedics, and 96.6 and 97.7% respectively reported to need more education or training in PPH. Moreover, 82.8% reported having managed patient(s) with PPH, but only 2.9% had performed AC. Prehospital personnels’ responses varied extensively regarding knowledge about what PPH is, how to estimate and handle PPH, and how to perform AC. Mean self-efficacy varied from 3.3 to 5.6, while collective efficacy varied from 1.9 to 3.8.

**Conclusions:**

This study indicates that prehospital personnel lack knowledge about PPH and AC, due to various responses to the developed questionnaire. Even though AC is an acknowledged intervention in PPH, few participants reported that this was utilized. Our findings emphasize the need for education and training in PPH and PPH handling generally, and in AC specifically.

**Supplementary Information:**

The online version contains supplementary material available at 10.1186/s12873-021-00490-8.

## Background

Postpartum hemorrhage (PPH) is a serious obstetric emergency, and one of the top five causes of maternal mortality globally [1]. Internationally, the prevalence of severe PPH appears to be rising, with increasing morbidity and need for transfusion therapy, and the mortality rates are high in low-income countries [2–6]. Maternal mortality rates (MMR) in the Nordic countries are among the lowest in the world, but women still die from complications of pregnancy or birth. In Norway, 168 maternal deaths were identified between 2005 and 2013 (Maternal Mortality Rate, MMR, 6.6 per 100,000), of whom 14 died due to severe PPH [7].

The incidence of PPH has been reported to vary between one to 5 %, depending upon the diagnostic criteria applied [2]. Although PPH is traditionally defined by the volume of blood loss observed, bleeding may not be visible externally or the blood may be mixed with amniotic fluid [4]. In 2017, the American College of Obstetricians and Gynecologists revised their definition of PPH to consist of the following criteria:

1. Cumulative blood loss ≥1000 ml or
2. Bleeding associated with signs or symptoms of hypovolemia within 24 h of the birth process, regardless of the route of delivery [8]

The most common causes of PPH include uterine atony, placental disorders, birth trauma and coagulation defects [9].

Timely diagnosis, appropriate resources and early management are critical to prevent death [9]. According to the World Health Organization, immediate solutions are needed to prevent women from dying of PPH [1]. Maternal mortality reviews have demonstrated that deaths caused by PPH are most likely to be preventable [10, 11]. The consistent application of a comprehensive protocol for management of PPH have been demonstrated to result in improved outcomes [12, 13]. Other potential interventions include tranexamic acid, fluid resuscitation, removal of the placenta, bimanual uterine compression, uterotonics, suturing of lower genital tract injury, blood product replacement, balloon tamponade, laparotomy, stepwise uterine de-vascularization, uterine compression sutures and hysterectomy. Emergency temporizing measures include application of the non-pneumatic anti-shock garment, uterine tourniquet application and aortic compression [14].

External, manual aortic compression (AC) is an emergency manoeuvre proposed to reduce postpartum haemorrhage and extend time for resuscitation and control of bleeding. The technique can be applied immediately to reduce bleeding from the uterus by reducing the blood supply. This again may prevent cardiac arrest from hypovolemia, and allow transfer to definitive care in-hospital. The Swedish obstetrician Bergstrom has been teaching this life-saving technique for many years in African countries, with great effect on maternal morbidity and mortality [15]. Nevertheless, in many countries, including Norway, manual AC is not actively used by healthcare personnel [16].

There is an increasing incidence of home births in Norway, as in Nordic countries [17, 18]. Hence, knowledge of PPH on competence in handling this condition is essential in prehospital personnel. Consequently, as researchers, healthcare personnel educators, nurse anesthetist and paramedic, our objective was to explore prehospital personnel’s knowledge about and self-assessed competence in PPH and AC, their experience with this condition, perceived need for more education and/or training, as well as their perceived self-efficacy and collective efficacy in PPH assessment and management.

## Methods

The study had a cross-sectional design, utilizing a questionnaire to assess prehospital personnel’s knowledge, self-assessed competence, self-efficacy and team-efficacy. The study adheres to the STROBE (Strengthening the reporting of observational studies in epidemiology) guidelines (see additional file 1).

### Setting and participants

The study was conducted in a county with approximately 317,000 inhabitants, within one hospital catchment area. There are five ambulance stations in this area. Prehospital personnel include ambulance assistants, ambulance workers (upper high school), bachelor in paramedicine or – nursing (180 European Credit Transfer and Accumulation System,

ECTs), and paramedics (further education, 60 ECTs). Utilizing a strategic sampling method, all prehospital personnel (*n* = 250) were invited to participate.

### Questionnaire

The questionnaire consisted of three parts:

1. a validated questionnaire in PPH self-efficacy (PPHSE) as well as PPH collective efficacy (PPHCE) [19]. The PPHSE includes eight items, using a continuous scale from 1 (never) to 8 (always). The items on self-efficacy focus on individual perception of control. The PPHCE includes 13 items (same scoring format as the PPHSE). In the current study, collective efficacy was defined as team-efficacy in the prehospital team, most commonly consisting of two healthcare personnel (see additional file 2.
2. a questionnaire developed as part of this study (see additional file 3)

In this study, the Cronbach’s alpha was 0.89 on the PPHSE scale, and 0.96 on the PPHCE, which is assumed excellent.

### Development of the questionnaire

Since no validated questionnaire to measure knowledge and self-assessed competence in PPH handling could be identified, we developed a questionnaire. Here, we used recommendations from the Delphi technique, which is suitable to obtain expert opinions in a systematic manner, and includes four steps: 1) expert input, 2) interaction with feedback, 3) statistical group responses, and 4) confidentiality [20, 21].

Experts were defined as specialists in their field, and included six anesthesiologists, three obstetricians and two midwives, knowledgeable in the field of obstetrics and obstetric anesthesia and recommended by other experts, four of them with prehospital experience [22]. The expert group consisted of four males, seven females, mean age was 53 years, and mean years of experience 15. In step 1 and 2, the expert group participated in the development of questions, and gave constructive inputs on clarity, wording, and contents of the whole questionnaire, as suggested by Streiner & Norman [23]. In these steps, experts received the questionnaire in two or three rounds depending on their inputs. In step 3, experts were asked to score the questionnaire regarding relevance, clarity and logic, on a scale from 1 = strongly disagree, to 5 = strongly agree. Table 1 presents the mean and range of responses to these scorings.

**Table 1:** Expert group scorings on the questionnaire

	a)	b)	c)	d)	e)	f)
Mean (range) I	3.3 (2–5)	3.6 (2–4)	4.6 (4–5)	4.1 (2–5)	3.9 (3–4)	3.9 (34)
Mean (range) II	4.3 (4–5)	4.3 (4–5)	4.8 (4–5)	4.6 (4–5)	4.6 (4–5)	4.5 (4–5)

Abbreviations: a) relevance to assess knowledge about PPH, b) relevance to assess competence in PPH handling, c) relevance to assess knowledge about manual aortic compression (AC), d) relevance to assess competence in performing AC, e) whether questions were clear, relevant and understandable, and f) whether the questionnaire was logic. 1 = strongly disagree, 2 = disagree, 3 = neither agree nor disagree, 4 = agree, 5 = strongly agree. I = first round, II = last round

The experts were involved in several rounds until consensus was reached. The final version of the questionnaire consisted of a) 13 knowledge questions with free-text answers, and two questions with alternatives yes/no/undecided, b) two questions about perceived need for more education and/or simulation, and c) five questions about experience with PPH and the use of AC.

### Analysis

Data were analyzed using the Statistical Package for the Social Sciences (SPSS 26.0) [24]. The descriptive statistics frequency, mean and Standard Deviation (SD) were used to analyse data. Internal consistency for the scales was tested by Cronbach’s alpha. There were no missing items in the validated questionnaires. The free-text responses were analyzed through a quantitative content analysis, reading through the responses and searching for similarities and code-words repeated throughout [25].

## Results

A total of 87 prehospital healthcare personnel (34.8%) responded to the questionnaire. Table 2 gives an overview of respondents’ gender, age, educational background and years of experience.

**Table 2:** Descriptives of the respondents (*n* = 87)

	n (%)
**Male gender**	50 (57.5)
**Age in years, mean**	37.9 (14.6)
**Age in years, range**	22–62
**Educational background n (%)**	
Assistant	6 (6.9)
Ambulance worker	41 (47.1)
Bachelor paramedicine	2 (2.3)
Bachelor nursing	4 (4.6)
Paramedic	29 (33.3)
Other	5 (5.7)
**Full time employee, n (%)**	71 (81.6)
**Years of experience, median (Interquartile range)**	12 (8–24)

Educational background; ambulance worker = upper grade school, Bachelor = 180 ECT (European *Credit* Transfer and Accumulation System), three years full-time, Paramedic = 30 ECT further education

### Responses to the developed questionnaire

#### Knowledge about PPH

On the question “How much is normal hemorrhage during birth, and when is it defined as postpartum hemorrhage?” most of the respondents assumed a hemorrhage of up to 500 ml as normal per-partum. Hemorrhage above 500 ml was interpreted as postpartum hemorrhage by 37 of the respondents. Other answers were ‘above 1 litre’ (*n* = 17), ‘2 l’ (*n* = 3), ‘1.5 l’ (n = 1) and ‘3–4 l’ (n = 1). The rest were undecided.

Regarding the question “How do you estimate the amount of hemorrhage during/after birth?” 39 respondents found this ‘difficult’. A total of 18 respondents reported to assess the sheets or diapers, how often they needed to be changed, or even to weigh them. In addition, 21 of the respondents reported to assess the patients´ vital parameters or level of consciousness.

#### Knowledge about interventions

Table 3 gives an overview of responses to the questions **“**Which interventions should be initiated in postpartum hemorrhage?” and “When you observe a life threatening hemorrhage, what do you do first?».

**Table 3:** Responses to questions about interventions that should be initiated and what prehospital personnel do first (n = 87)

	Which interventions should be initiated in postpartum hemorrhage?, n=	When you observe a life threatening hemorrhage, what do you do first? n=
Uterus massage	29	27
Fluid resuscitation	22	24
Put the baby to breast	14	12
Abdominal massage	10	8
Establish venous access	9	14
Add pressure on the abdominal aorta	9	9
Elevate legs	9	9
Quick transport to hospital	8	14
Oxygen treatment	7	9
Put pressure on abdomen	5	5
Shock treatment	5	6
Put the fist into the woman and add pressure from the inside	5	5
Areola massage	4	1
Hemorrhage control	4	8
Oxytocin	3	3

Other suggestions interventions were ‘add pressure on the inguinal aorta’, ‘analgesia’, ‘tranexamacid’, ‘comfort the mother’, ‘compression’, ‘early warning to the hospital’.

Other “Clinical situations than postpartum hemorrhage where manual aortic compression can be lifesaving» reported were ‘hemorrhage in the lower extremities” (*n* = 7), ‘abdominal aorta aneurism’ (AAA) (n = 7), ‘other vaginal hemorrhage’ (*n* = 6), amputations (*n* = 4), other causes of massive hemorrhage such as extrauterine pregnancy (*n* = 2), and open wounds (n = 2).

Whether the ambulance had any drugs for use in situations of postpartum hemorrhage, most respondents reported ‘no’ (82.8%), while 11.5% were undecided, and 5.7% of the respondents reported ‘yes’. Drug reported accessible was oxytocin, and side-effects of this drug was reported to be ‘high blood-pressure’ (*n* = 1), and ‘nausea and vomiting’ (*n* = 1).

To the question “When is manual aortic compression (using a fist on aorta) appropriate?”, responses were ‘in massive hemorrhage’ (*n* = 32), ‘in PPH’ (*n* = 13), ‘in life-threatening hemorrhage’ (*n* = 5), ‘when the child is delivered’(*n* = 2), ‘when uterus massage does not have an effect’ (n = 2), and ‘AAA’(n = 1) (non-response, n = 32).

Contra-indications to AC reported were ‘limited hemorrhage’ (*n* = 28), ‘child not delivered’ (*n* = 6), ‘pain’(n = 1), and ‘patient awake’ (n = 1) (non-response, *n* = 51).

#### Knowledge about performance of AC

When asked «How would you provide manual aortic compression?» 12 of the respondents reported ‘establish pulse in arteria femoralis, add pressure above the uterus until absence of pulse’. And 20 respondents reported to ‘add pressure on the abdomen’, but location of pressure varied from ‘under the diaphragm’, ‘umbilical area’, or ‘in the middle’. Ten respondents answered ‘add pressure on the aorta’, four respondents reported ‘add pressure both from the inside and outside’, and three ‘add vaginal pressure’.

When asked what the purpose of AC is, 71 respondents reported ‘to stop the hemorrhage’. On the question “How do you assess whether the maneuver is conducted correct?”, 35 responded ‘when the hemorrhage stops’, and 18 responded ‘when the pulse in arteria femoralis is absent’. Regarding considerations during drug administration and ongoing AC, five respondents reported ‘side-effects’, and one reported ‘that drugs are not transported beyond the location of pressure’. A total of 82.8% of the respondents reported ‘no’, 11.5% reported ‘undecided’ and 5.7% reported ‘yes’, to the question about whether there are potential complications related to AC. Suggested complications were ‘damage due to ischemia’ (*n* = 4), ‘reduced blood pressure’(*n* = 3), ‘damage to inner organs’(*n* = 1), and ‘pain’ (n = 1).

No relation between educational background and level of knowledge could be identified.

### Need for education and/or training

When asked “Do you want more education in handling postpartum hemorrhage?”, 96.6% responded ‘yes’, 1.1% responded ‘no’, and 2.3% responded ‘undecided’.

Among the respondents, 97.7% answered ‘yes’ that they want more training/simulation in handling postpartum hemorrhage.

Participants that responded ‘no’ or ‘undecided’ that they needed more education or training were all assistants or ambulance workers.

### Experience

Prehospital personnel’s experience with PPH and AC is shown in Table 4.

**Table 4:** Experiences with PPH and AC (n = 87)

	n (%)
Have experience with PPH	72 (82.8)
Have used AC	2 (2.3)
Have considered using AC	5 (5.7)
Had patients where AC may have been appropriate	
Yes	6 (6.9)
No	70 (79.3)
Undecided	11 (13.8)

AC = external, manual, aortic compression

Reasons for not using AC were ‘lack of education’ (74.7%), ‘lack of training’ (10.3%), ‘feel unsecure on the procedure’ (10.3%), and ‘difficult to cause the patient pain’ (4.6%) (fixed response alternatives).

### Self-efficacy in PPH

Table 5 gives an overview of the responses to the PPH self-efficacy questionnaire (PPHSE).

**Table 5:** Self-efficacy in PPH (n = 87)

	Mean score, standard deviation in parenthesis
I remain calm when handling PPH	5.5 (2.1)
I have experienced being able to act in situations with PPH	2.6 (1.5)
I can handle PPH whenever it happens	4.4 (2.1)
I can carry out the necessary actions to handle PPH	4.7 (2.1)
I am confident in how to treat PPH	3.5 (2.6)
I am able to stay calm in emergency situations	4.1 (2.2)
I am able to identify PPH at an early stage	3.6 (2.0)
PPH will make me feel paralyzed/unable to act	4.2 (2.2)
Total score	4.0 (1.6)

PPH = postpartum hemorrhage. Scored on a scale ranging from 1 = never to 8 = always

Table 6 gives an overview of the responses to the PPH collective efficacy questionnaire (PPHCE).

**Table 6:** Collective efficacy in PPH (n = 87)

	Mean score, Standard deviation in parenthesis
As a team, we help each other prevent excessive PPH	6.9 (1.4)
As a team, we are able to carry out the necessary actions to treat PPH	5.7 (2.4)
I think the team will share tasks in an appropriate way during PPH	6.5 (1.7)
The team can handle PPH	5.0 (2.1)
I think that every member of the team will express themselves clearly during PPH	4.8 (2.0)
As a team we can cope with PPH	5.6 (2.1)
The team usually has clear leadership in emergency situations like PPH	6.1 (1.7)
When PPH arises, our team is able to take action	5.1 (2.2)
As a team we communicate clearly and efficiently whenever PPH arises	5.5 (2.1)
Everyone knows what to do during an ongoing PPH situation	5.9 (1.9)
We are able to identify PPH at an early stage	5.9 (2.0)
We as a team remain calm during situations involving PPH	5.3 (2.0)
We are supportive of each other when we are in high-pressure situations	5.6 (2.0)
Total score	5.6 (1.6)

PPH = postpartum hemorrhage. Scored on a scale ranging from 1 = never, to 8 = always

## Discussion

Our findings indicate that prehospital personnel lack knowledge about postpartum hemorrhage (PPH) and manual aortic compression (AC). As much as 82.8% had experienced PPH, but only 2.3% had utilized AC. Participants scored lower on self-efficacy than on collective efficacy in PPH handling. 96.6 and 97.7% respectively reported a need for more education or training in PPH/PPH handling.

Our findings enlighten the knowledge gap in prehospital personnel regarding PPH and AC. This indicates a need to include this topic in educational programs regardless of educational level. Studies have indicated a need to develop and implement robust clinical research regarding treatment of PPH, to establish an international knowledge platform [26]. Senthiles et al. [27] emphasize a need to reach a broad consensus about the most efficient interventions to prevent and treat PPH. This include PPH prevention initiatives, estimation of blood loss, when and which uterotonica to administer, and use of blood products. Nevertheless, consensus has been reached regarding the main steps for the initial management of PPH: manual exploration of the uterus, visual assessment of genital tract, bladder indwelling catheter, measures to maintain maternal temperature, supplemental oxygen, uterine massage, maintenance of venous access, infusion of crystalloids rather than colloids, continuous monitoring of pulse, blood pressure and respiratory rate, and use of a uterotonic [27].

The participants’ self-efficacy was scored lowest on the items «I have experienced being able to act in situations with PPH” (mean score 2.6, SD = 1.5) and “I am confident in how to treat PPH” (mean score 3.5, SD = 2.6), while the highest score was on the item “I remain calm when handling PPH” (mean score 5.5, SD = 2.1). In contrast, the mean score on the item “I am able to stay calm in emergency situations” was 4.1 (SD 2.2). This may indicate that prehospital personnel feel self-efficient when handling more stable PPH-patients, but not in emergency PPH. Prehospital personnel is used to handling acute and unpredictable situations, hence this question has to be seen in relation to PPH-handling, and not as a general approach to emergency situations. The lowest score regarding collective efficacy was on the item “I think that every member of the team will express themselves clearly during PPH” (mean score 4.8, SD = 2.0), and the highest score on the item “As a team, we help each other prevent excessive PPH” (mean score 6.9, SD = 1.4). This may mostly reflect the nature of prehospital personnel’s work, rather than PPH itself [28]. In contrast, a recent study including pre- and post-simulation scores of self- and collective efficacy in PPH in intra-hospital personnel, participants scored 5.9 (SD = 1.1) on self-efficacy before the simulations, and 6.5 (SD = 0.9) after. Scores on collective efficacy in PPH handling increased from 5.8 (SD = 0.9) to 6.3 (SD = 0.8) [19]. This may indicate that personnel working in obstetric/resuscitation teams in hospital are more trained in this obstetric emergency, which is also natural.

Aronson and Bergström’s research [15] indicates that AC may effectively reduce need for blood transfusion, morbidity and mortality. Nevertheless, our participants rarely used this technique. This study, as well as earlier research enlighten the challenges in distributing knowledge to larger groups of personnel, and from high-income to low-income countries. Hence, a PPH digital learning program was developed, in addition to a simulator that enables participants to evaluate the pressure, placement and effect of AC. After the conduction of this study, prehospital personnel attended the course and were given a demonstration and opportunity to apply AC. In addition, a new PPH handling guideline has been implemented in hospital, resulting in that midwives and obstetricians now often arrive to the operating room in the patient’s bed, adding AC. In Tanzania, a structured inter-professional simulation program on PPH handling lead to a significant reduction in the use of ≥5 units of blood products related to severe bleeding after birth [19]. Moreover, training that included all levels of maternity staff, repeated sessions with realistic scenarios, and debriefing contributed to reduced blood transfusion rates in this high-risk maternity settings [29]. The same research team conducted a study on the effect of inter-professional simulations to reduce PPH, in a Norwegian university hospital. The researchers emphasized the importance of team training as a learning feature, and that inter-professional simulation enhanced self-efficacy and reduced perception of stress. Here, personnel also experienced an improved competence to provide efficient PPH management [30].

It may be argued that research conducted in low-income countries (LIC) are not transferable to high-income countries (HIC) similar to the setting for this study. Moreover, it may be argued that the study presented here is not as relevant due to the low incidence of PPH in HIC settings. Still, there is an increasing incidence of home births in Norway, as in Nordic countries [17, 18], and we argue that knowledge of PPH on competence in handling this condition is essential in prehospital personnel. Self-assessment of knowledge, experiences, teamwork and need for more education and training provide possibilities to provide tailored training and education of healthcare personnel, also in rare conditions.

### Limitations

Due to the small sample size, findings here may not be generalizable to other countries or settings. Nevertheless, research support our findings, stating a need for more knowledge and competence in PPH and PPH handling internationally. Two validated tools were used to measure self-efficacy and collective efficacy, which increase the reliability of the study. Moreover, the new questionnaire was developed in-line with recommendations from the Delphi technique, and face- and content validity were high. The eight point scale may fail to measure the true attitudes of respondents. Also, it is not unlikely that responses will be influenced by previous questions. In the current study, there may have been some mis-interpretations of items on the PPHSE and PPHCE due to that the PPH reference was not included on all items.

## Conclusion

Our findings indicate that prehospital personnel lack knowledge about postpartum hemorrhage (PPH) and manual aortic compression (AC). Hence, this study indicates a need for more education and training in PPH and PPH handling in prehospital personnel, to be able to assess and manage PPH.

### Implications for further research

Responses on the free-text questionnaire in this study will be used to develop a tool with fixed response alternatives, for assessment of healthcare personnel’s knowledge about PPH and AC that can be used in other settings to assess knowledge gaps and what to focus on in quality improvement initiatives.

## Supplementary informations

**Additional file 1.** The STROBE (Strengthening the reporting of observational studies in epidemiology) checklist.

**Additional file 2.** The postpartum haemorrhage self-efficacy (PPHSE) and -collective efficacy (PPHCE) items.

**Additional file 3.** The questionnaire developed as part of the study, assessing knowledge and self-assessed competence in PPH handling.

**Additional file 4.** Anonymized data.

## Data Availability

All data generated or analyzed during this study are included in this published article, and is attached as a supplementary information file (see additional file 4).

## Abbreviations

AAA: abdominal aorta aneurism
AC: aortic compression
ECT: European Credit Transfer and Accumulation System
PPH: postpartum haemorrhage
PPHCE: postpartum haemorrhage collective efficacy
PPHSE: postpartum haemorrhage self-efficacy
SD: standard deviation
