# Supplementary material for: Prehospital assessment and management of postpartum haemorrhage- healthcare personnel’s experiences and perspectives
Source: BMC Emerg Med. 2021 Aug 28;21:98. doi: 10.1186/s12873-021-00490-8 (PMC8403351; doi:10.1186/s12873-021-00490-8)
Supplement: Supplementary file 2 — Additional file 2. The postpartum haemorrhage self-efficacy (PPHSE) and -collective efficacy (PPHCE) items. [file 12873_2021_490_MOESM2_ESM.docx]

**Additional file 2**

**Questions in the postpartum hemorrhage self-efficacy (PPHSE)**

Scoring: eight-point Likert scale that range from 1 (never) to 8 (always).

1. I remain calm when handling PPH
2. I have experienced being able to act in situations with PPH
3. I can handle PPH whenever it happens
4. I can carry out the necessary actions to handle PPH
5. I am confident in how to treat PPH
6. I am able to stay calm in emergency situations
7. I am able to identify PPH at an early stage
8. PPH will make me feel paralysed/unable to act

**Questions in the postpartum hemorrhage Collective efficacy (PPHCE)**

Scoring: eight-point Likert scale that range from 1 (never) to 8 (always).

1. As a team, we help each other prevent excessive PPH
2. As a team, we are able to carry out the necessary actions to treat PPH
3. I think the team will share tasks in an appropriate way during PPH
4. The team can handle PPH
5. I think that every member of the team will express themselves clearly during PPH
6. As a team, we can cope with PPH
7. The team usually has clear leadership in emergency situations like PPH
8. When PPH arises, our team is able to take action
9. As a team, we communicate clearly and efficiently whenever PPH arises
10. Everyone knows what to do during an ongoing PPH situation
11. We are able to identify PPH at an early stage
12. We as a team remain calm during situations involving PPH
13. We are supportive of each other when we are in high-pressure situations
