# Supplementary material for: Prehospital assessment and management of postpartum haemorrhage- healthcare personnel’s experiences and perspectives
Source: BMC Emerg Med. 2021 Aug 28;21:98. doi: 10.1186/s12873-021-00490-8 (PMC8403351; doi:10.1186/s12873-021-00490-8)
Supplement: Supplementary file 3 — Additional file 3. The questionnaire developed as part of the study, assessing knowledge and self-assessed competence in PPH handling. [file 12873_2021_490_MOESM3_ESM.docx]

**Knowledge and competence in prehospital post-partum handling**

Postpartum hemorrhage (PPH) is a serious obstetric emergency, and one of the top five causes of maternal mortality globally. The most common causes of PPH include uterine atony, placental disorders, birth trauma and coagulation defects. Even though the incidence is higher in low-oncome countries, this is also increasing in Norway. Identification and immediate action are essential to prevent increasing morbidity, need for blood transfusions, and in worst case, death.

**Background**

1. **What is your educational background? (several options possible)**

| Ambulance- assistant |  |
| --- | --- |
| Ambulance worker (Upper High School) |  |
| Bachelor paramedicine/paramedic |  |
| Bachelor nursing |  |
| Paramedic further education |  |
| Other (please write with your own words) |  |

**2. Years of experience from current workplace?** (number of years)

**3. Years of experience from working in ambulance, in total?** (number of years)

_______________

**4. Is your employment permanent?**

| **Yes** | **No** | **Part time (% position)** | **On-call** |
| --- | --- | --- | --- |
|  |  |  |  |

**5. Gender:** Male Female

**6. Age (number of years)**

**7. Where do you work (which ambulance station)?**

**Knowledge**

**8. How much is normal hemorrhage during birth, and when is it defined as postpartum hemorrhage? (please describe in your own words)**

**9. Which interventions should be initiated in postpartum hemorrhage? (please describe in your own words)**

**10. How do you estimate the amount of hemorrhage during/after birth? (please describe in your own words)**

**11. When you observe a life threathening hemorrhage, what do you do first? (please describe in your own words)**.

**12. Are there other clinical situations than postpartum hemorrhage where manual aortic compression can be lifesaving? (please describe in your own words)**

**13. Do you have any drugs for use in situations of postpartum hemorrhage in the ambulance?**

**Yes No Undecided**

**If yes, which ones? (please describe in your own words)**

**What are the side-effects of these drugs? (please describe in your own words)**

**14. When is manual aortic compression appropriate? (please describe in your own words)**

**15. When is manual aortic compression not appropriate? (please describe in your own words)**

**16. How would you provide manual aortic compression? (please describe in your own words)**

**17. What is the intention with manual aortic compression? (please describe in your own words)**

**18. How do you assess whether the manouvre is conducted correct? (please describe in your own words)**

**19. Which considerations do you do regarding drug administration during manual aortic compression? (please describe in your own words)**

**20. Are there any complications related to manual aortic compression?**

Yes No Undecided

**If yes, which complication? (please describe in your own words)**

**Knowledge- and competence needs**

**21. Do you want more education in handling postpartum hemorrhage?**

| **Yes** | **No** | **Undecided** |  |  |  |
| --- | --- | --- | --- | --- | --- |
| **□** | **□** | **□** |  |  |  |

**22. Do you want more training/simulation in handeling postpartum hemorrhage?**

| **Yes** | **No** | **Undecided** |  |  |  |
| --- | --- | --- | --- | --- | --- |
| **□** | **□** | **□** |  |  |  |

**Experience**

**23. Do you have any experience(s) with postpartum hemorrhage?**

| Yes | No Undecided |
| --- | --- |
| □ | □ □ |

**24. Have you ever used manual aortic compression on a patient?**

| Yes | No Undecided |
| --- | --- |
| □ | □ □ |

**25. Have you ever considered using manual aortic compression on a patient?**

| Yes | No Undecided |
| --- | --- |
| □ | □ □ |

**26. Have you ever had patients with massive hemorrhage where you in retrospect see that you could have used manual aortic compression?**

| **Yes** | **No** | **Undecided** |
| --- | --- | --- |
| **□** | **□** | **□** |

**27. What was the main reason for not conducting manual aortic compression?**

**Lack of education Lack of training Uncertainty Difficult to cause pain/discomfort**
