# Supplementary material for: Prehospital assessment and management of postpartum haemorrhage- healthcare personnel’s experiences and perspectives
Source: BMC Emerg Med. 2021 Aug 28;21:98. doi: 10.1186/s12873-021-00490-8 (PMC8403351; doi:10.1186/s12873-021-00490-8)
Supplement: Supplementary file 4 — Additional file 4. Anonymized data. [file 12873_2021_490_MOESM4_ESM.pdf]

@_id	formid	var1	Utdanning	Erfaring_ar	Erfaring_ar	Fast_ansatt	Kjønn	Ambulanse
8936635	156715	45	6	3	22	2	2	
9010727	156715	47	4	20	24	1	1	2
9010839	156715	54	5	27	27	1	1	1
9011584	156715	29	5	8	11	1	2	1
9011699	156715	26	2	10	12	1	1	5
9012299	156715	37	5	13	13	1	2	5
9013659	156715	43	2	3	3	1	1	1
9015986	156715	43	2	2	2	2	1	2
9015849	156715	49	2	20	20	1	2	5
9026689	156715	56	2	8	12	1	2	3
9026690	156715	24	2	6	6	1	2	3
9027898	156715	52	5	27	27	1	1	
9029437	156715	30	2	8	7	1	2	5
9029470	156715	26	3	2	2	1	1	5
9057877	156715	23	2	1	5	1	1	5
9057176	156715	30	2	3	10	1	2	3
9057781	156715	50	5	23	23	1	1	4
9058189	156715	31	5	12	12	1	2	2
9058134	156715	30	2	3	3	1	2	5
9085719	156715	49	2	10	10	1	2	3
9113348	156715	37	5	17	17	1	1	5
9120134	156715	26	6	1	1	1	2	5
9123949	156715	61	1	0	0	4	2	5
9132041	156715	37	5	14	14	1	2	3
9139167	156715	56	2	10	10	1	2	2
9141917	156715	29	2	8 år	8 år	1	2	1
9141920	156715	25	2	2år	2år	3	1	1
9144206	156715	41	5	5	5	1	2	5
9178265	156715	36	5	10	10	1	1	3
9191339	156715	47	6	24	24	1	1	3
9193069	156715	42	2	16	16	1	1	2
9199050	156715	40	5	18 år	13 år	1	2	3
9199451	156715	28	5	8	10	1	2	1
9230620	156715	44	6	1	1	2	1	4
9231109	156715	62	2	4	4	1	1	2
9231524	156715	33	2	5	5	1	2	2
9251292	156715	49	5	25	25	1	1	3
9255205	156715	56	4	9	35	1	1	4
9257565	156715	51	5	25	25	1	1	4
9269348	156715	56	5	24	24	1	2	3
9286078	156715	35	5	9	14	1	1	6
9288433	156715	29	4	7	7	1	1	2
9287745	156715	56	5	8	8	1	1	1
9289497	156715	32	5	11	14	1	1	4
9289450	156715	38	2	20	20	1	1	4
9291815	156715	26	2	6	6	1	1	1

9307197	156715	49	1	6	6	4	1	4
9306437	156715	29	2	6	6	1	1	2
9316139	156715	28	2	6	6	1	1	4
9314141	156715	31	3	0	1	3	2	1
9316376	156715	36	1	8	8	1	1	3
9321624	156715	24	2	6	6	1	1	2
9326267	156715	37	5	10		1	1	3
9341692	156715	24	1	1	1	2	2	5
9385809	156715	44	5	24	24	1	1	5
9425311	156715	24	2	6	6	1	1	4
9456036	156715	32	1	1	1	2	2	5
9463893	156715	29	4	1	1	4	2	4
9463073	156715	32	2	4	4	1	2	2
9465748	156715	50	5	24	28	1	1	1
9466726	156715	37	6	5	5	4	1	4
9467645	156715	48	1	2	2	4	1	5
9466624	156715	49	2	7	7	1	2	1
9469155	156715	46	2	7	7	1	2	5
9469549	156715	36	2	12	12	1	1	5
9468764	156715	27	2	6	6	1	2	5
9469641	156715	40	2	8	8	1	1	5
9486702	156715	36	5	6	12	1	1	1
9486871	156715	22	2	4	4	1	2	1
9486672	156715	24	2	2	2	3	2	1
9488559	156715	26	2	8	8	1	1	1
9486494	156715	39	5	20	20	1	1	1
9486640	156715	27	2	2	2	3	2	1
9486657	156715	26	2	8	8	1	1	1
9488569	156715	48	2	3	3	1	1	1
9488777	156715	28	2	8	10	1	1	1
9477283	156715	50	5	17	23	1	1	4
9500808	156715	53	2	25	30	1	1	4
9509409	156715	33	2	12	12	1	2	4
9549290	156715	45	5	15	15	1	2	1
9558512	156715	50	5		30	1	2	5
9569159	156715	22	2	10 månede	3	1	2	5
9614669	156715	46	5	24	24	1	1	1
9687629	156715	40	2	4	6	1	2	5
9726507	156715	49	5	8	25	1	1	2
9908351	156715	43	2	2	2	3	1	2
9969265	156715	36	5	6	18	3	1	1

Var9	var10	var11	var12	var13	var14	var15	var16	var17
vet ikke	vet ikke							
500-750 m	Uterus mas	Noe vanske	Stanse blø	Store skade	2		TXA, mest a	Benyttes ve
halv liter	blødningsk	Monitorere	manuell ao	aortadissek	2			Ved stor liv
Etter en va	Om mor bl	Se hvor my	Be om bist	Ved store a	2	Nei, ingen	Ingen nevn	Ved postpa
Ikke mer e	Massering	"Ballpark"	Be om bist	Skader i be	2			Postpartum
0,5 liter	massere/k	Jeg syntse	Be om bistand, helst h		2			
ca. 500ml	stoppe blø	må se visue	Forsøke å s	Ved hjertes	2			Når man se
Opptil en h	Massere ut	Har aldri va	Bistand fra	Kjenner ikk	2			Kjenner ikk
Opp mot e	Presse med	Man ser jo	Prøve og st	Alle store b	2			Når de blør
Opp mot 5	Vi må legge	Vi ser på m	Bolusdose	Vet ikke.	2			Når blødnir
noe blødni	venekanyle	mengde bl	bolusdose	AAA mulig	2			ved blødnir
Noe blødni	Massasje a	Dette er va	Utvendig m	Usikker, uk	2			Vet ikke, m
I gjennoms	Først må vi	Det er ikke	Forsøke å s	Om placen	2			Ved en stør
pågående b	Bør masser	Det skal bl	Det man al	XU ?	2			Hvis ungen
Usikker, m	Barn mot b	Vil følge m	Vil først og	Siden dere	2			Ved stor bl
Postpartum	Spørs hvor	Det synes j	Være rask	Aortaaneu	1	Nacl	nei	Ved stor på
Jeg tenker	Etablere Iv	Bevissthet-	Stanse blø	Åpne blødr	2			Åpen blødr
500 ml er r	Massere br	Hvor ofte e	Heve bena,	Store kutt,	2			Når du ikke
500ml norr	volumbeha	ut i fra mer	aortakomp	usikker	2			ved alvolig
500 ml Me	Væsketilfø	Normal blø	Aortakomp	Usikker	1	Væske og oksygen		Ved alvorli
					2			
Vet ikke	Usikker	Usikker	Usikker	Usikker	3		Vet ikke	Ved blødni
Vet ikke	Press på ac	Vet ikke al	Vet ikke	Trippel A?	3			Vet ikke
500 ml pos	væskebeha	tilstanden	forsøke å s	vet ikke..	2			ved truend
Noe blod n	Prøve å he	Noe blod i	Prøve å he	Hvis aorta	2		Det regner	Tenker at
500ml, når	Kjør fort til	Jeg vet ikke	Legge inn P	Ved skader	2		Vet ikke	Vet ikke
1Liter	konferer m	??	heve bein og få inn ve		2			
Opptill 500	Legge barn	Vanskelig,	Bistand! Komprimere		2			Ved livstru
0,5L liter e	Legge barn	Om mulig s	Sikre grove venøse inn		2			Ved alvorlig
Lærebøker	Legge pas f	Vurdere m	Vurdere on	Stor vahina	2		Blodtrykksf	Dersom ing
0,5 liter er	livmor mas	Dette er va	Iv tilgang, væske,aorta		2			Etter fødse
Er usikker	Uterusmas	Om det er	uterusmass	Traumatisk	2			Ved stor bl
normalt: in	ABC, oksyg	vanskelig å	tilkalle assi	store blødr	2			
Det er norr	Massere liv	Jeg ville ha	Vurderer pasientens kl		2			Når tiltak s
normalt å b	når man sk	Kan være v	stanse blødning ved hj		2	ingen	ingen	når det ikke
ca 400-600	livmormass	vanskelig å	Prøve å stoppe/reduse		2			Når man sk
Ca halv lite	Massere livmoren ved å trykke mot øvre del a				2			
Pas har en	Vid väldig k	Väldigt svå	Aorta tryck	Misstänkt t	2			Når pas ha
varierende	heve beina	Etter beste	hev beina , start væske		2			når pas har
Normalt å l	PVK tilgang	Det er vans	Abdominal	Traume mc	1			Ved store,
500ml, ov	Heve beina	Har ingen v	ABCDE	Vet ikke	2			
					2			
ca 400 til 6	bytte kladd	det er vans	livsmormas	kompresjo	2			ved fortsat
500 ml om	stoppe eve	500 - 1000	stoppe blø	tryke hart p	2	Veske behandling ved		pågende st
Det er norr	Vanlige tilt	samle blod	massere livmor. volun		2			ved stor på
0,5 liter	Man kan massere og k		må prøve å legge press		3			Når blødnir

Opp i mot :	Kan masser	Friskt blod	Stor pågåe	Store blødr	2			Når vi føler
500ml, ane	Hvis stor bl	Med sivenc	Gi oksygen, to grove v		2			
500ml er n	Stoppe blø	Ukjent	Be om bist	Åpne skade	2	Har ikke	Har ikke	Når det ikk
Det er norr	Uterus mas	Det er norr	Tilkalle bistand og vars		2			Ved livstrue
					2			
5 dl	Iv inngang,	Det jeg kan	Press mot a	Usikker	2			Ved livstru
Det er ikke	Masere ma	Ser etter m	Aortakomp	Ved store b	2			Store blødr
Inntil ca 50	1-2 grove p	Det synes j	Jeg ville rol	Ikke som je	2	Oksygen og	O2 kan ha	Ved livstru
Ikke sikker,	Usikker	Antall bind	Be om bist	AAA	2			Usikker
500 ml skal	Forberede	Dette er va	Manuell ko	store skade	2			Stor blødni
300ml er g	Hard komp	Det er vans	Vet ikke	Vet ikke	3	Oksytocin	Oksytocin	Når blødnir
Det er norr	Legg inn gr	Måler vital	Bimanuell	Andre blød	3			
over 500 m	Legge barn	Ser værre	Stanse den	Vet ikke..	2			Når pasient
En halv til	Massere o	Skjønnsme	Heve ben c	Vet ikke.	2			
1 liter	Symtombeahandle , va		Rask transp	Ved høy an	3			Når det er
Inntil en ha	Massere ute	Prøve å da	Be om støtte fra jordm		2			Når morkak
Normal blø	Dersom mo	Dette er va	For at vi sk	Blødning i l	2			Ved Postpa
Vet ikke		Usikker, m	Vet ikke		2			
Blødning fø	Man gir et	Vurderer s	Usikker på	Usikker	3			
					2			
Ca 500 ml	Iv inngang	Har ingen e	Komprimere over livm		2			
500 ml	Hurtig trsp	Vanskelig	Stanse blø	Livstruende	2			Ved stor bl
500ml - 1l	Heve ben,	Vanskelig å	Væskebolu	Store blødr	2			Stor blødni
	Blødningskontroll		Manuell aortakompres		2			
Blødning et	Heve ben.	Massiv blø	Heve ben	Usikker	3	..	..	Massiv blø
					3			
2L	Massere or	Bevissthet	Få hjelp :)	Usikker	2			Når det er
Vet ikke	Usikker Ha	Fra egen er	Prøver og k	Vet ikke	2			Usikker
Normalt å l	Sjokktiltak	Estimat pre	Stoppe blø	Når placen	3			Etter fødse
0,5 L	Stimuli på	Vanskelig d	Be om ress	Nei	2	Urelevant	Urelevant	
Blødning o	Trykk på ut	Med erfari	Trykk på utrus, evt gå i		2			Ikke effekt
0,5 l	heve beina	anta sånn	heve bein	hj frek und	2			
Ganske no	sjokkleie o	Antar ca m	komprimere uterus utv		2			
Normalt o	Massasje a	Vanskelig s	Massere liv	Ved livstru	2			Ved livstrue
normal blø	massere liv	sammenlik	be om bist	rifter, hem	2			når det er s
Det er norr	Det finnes	Det er vanskelig å vurdere en blød			2			
Over 1 liter	Stanse blø	Ser hvor m	Kompresjon		2			Blødning et
Ca. 500ml	Kompresjo	Erfaring. Si	Komprimer	Rift i livmo	2			Der det er s
alt over 50	massering	vanskelig s	be om bist	usikker. sto	1	skal brukers av jordmo		ved kraftig
4-600ml	Livmormas	Veie bleie	Be om bist	Tenker at n	2		Har ikke m	I forbindels
Opptil 500	Massere liv	Vanskelig å	Masere utv	Traumer i	2	Tidl arbeids	Syntocinon	Kritisk blød

var18	var19	var20	var21	var22	var23	var24	var25	var26
Når man ko	Med krafti	Å reduser b	Sjekk og lol	Usikker	3		1	1
Ukjent	med to kny	Stenge av a	se at blødn	Vet ikke, da	3		1	1
Når det ikk	Direkte kor	Minimere/s	Se om blød	Vi har inge	3	Er vel alltid	1	1
Når det ikk	Vet ikke.	Minimere t	Vurdere bl	Vet ikke. Kj	3		1	1
		begrense blødning					1	1
r at det er p	Bruke knyt	for å stopp	Se om blødningen stop		3		1	1
Kjenner ikk	Kjenner ikk	Kjenner ikk	Kjenner ikk	Kjenner ikk	3		1	1
Hvis de ikke	Trykke kny	stoppe blø	Slippe opp og se om de		1	Skade på u	1	1
Før barnet	Knyttneve	Å stoppe bl	Stopper blødningen ha		3		1	1
når barnet fortsatt er i		stoppe blø	at blødning	bivirkninge	3		1	1
Vet ikke	Vet ikke he	Klemme av	Pulsbortfal	Vet ikke	3		1	1
re blødning	Aldri hatt o	Stanse/ mi	Det kan man se på me		3		1	1
Hvis ungen	Klemme ne	Stanse så n	Kjenne på l	eventuelle	3		1	1
Når pasient	En hånd fø	Forhindre s	Om blødningen minske		3	Så ikke noe	1	1
Når det ikk	press med	Hensikten	Om det sto	At ved ønsl	3		1	1
Indre blødn	Legge høyt	Reduser ell	Sjekke puls	Bivirkninge	1	Vevsdød	1	1
?	Trykke kny	Klemme av	Vil se det d	Så sant det	3		1	1
usikker	trykk knytt	stoppe blø	se til at blødningen ha		3		1	1
Usikker	Press mot a	Stoppe blø	Blødning a	Usikker	3		1	1
					3		1	1
Vet ikke	Vet ikke	Stoppe blø	Vet ikke	Vet ikke	1	Usikker	1	1
Vet ikke	Vet ikke	Vet ikke	Puls i femoralis?		1		1	1
vet ikke	klemme på	minske blø	vet ikke	vet ikke	3	vet ikke	1	1
Ved våken	Med hardt	Helst stopp	Blødning a	Manuell ao	1	En stenger	1	1
Vet ikke	Vet ikke	For å stopp	Se om det	Vet ikke	3		1	1
vet ikke	vet ikke	for å stopp	ser om blø	vet ikke	3		1	1
Livreddend	Direkte kor	Stoppe blø	Klinikk		3		1	1
ge blødning	press med	Klemme av aorta til livmor.			3		1	1
Når andre t	Palpere pul	Direkte tryk	Reduksjon	Riktig vene	1	Muligens d	1	1
vet ikke	setter en k	stoppe elle	lyskepulser	vet ikke	3		1	1
Når man ha	Med sterkt	stoppe blø	Om du slut	usikker	3		1	1
hvis noen e	lage en kny	stoppe/der	blødningen avtar		1	ved aortak	1	1
om masseri	Jeg har en l	Er å stoppe blødningen i livmoren.			3		1	1
når det ikke	da skal ma	stanse stor	se om blød	gi smertest	2		1	1
når blødnin	presser kny	Stoppe blø	redusert eller opphørt lyskepuls, redusert syn				1	1
					3		1	1
Skulle pers	Pas ligger p	Stänga av c	Är väldigt s	Morfin sen	2		1	1
ett betydel	man finner	få blodning	kjenne på pulsen i lysken om den har blitt sva				1	1
Skal ikke be	Man øver e	Stanse blø	Blødningen	Vi har ikke	1	Organskad	1	1
					3		1	1
					3		2	1
t blødning e	finne lyske	redusere bl	redusert el	gi forvarme	3		1	1
?	tryke hart på magen, n		?	dette lærer	2		1	1
gående blø	Plasser kny	forhindre s	sjekk lyskepuls. kjenne		3		1	1
Det skal ikk	knyttneven	Legge press på blødningen, prøve			1	det vil være	1	1

Når klinikk	Knyttet nev	Stanse/beg	Bortfall av	En stor del	1	Vevsskade	1	
		Stopp blodtilførsel, og med det st			3		1	1
Når man ha	Usikker, me	Stoppe livs	At blødning	Ukjent	1	Utilstrekke	1	1
ende postpa	Press knytt	Legge press	At blødningen stopper		3		1	1
					3		3	3
Det vet jeg	Det vet jeg	Kutte av bl	Det vet jeg ikke		1	Legger tryk	3	3
Der hvor en	Aortakomp	Begrense b	Lyskepuls	Vet ikke.	3		1	1
Alle tilfeller	En hånd pr	At pasiente	Jeg vet ikke	At det burc	3		1	1
Usikker	Usikker	Hindre sjok	Bedring i vi	Usikker	3		1	1
Når blødnin	Lag en knyt	stoppe blod	Sjekke fem	usikker	1	Skade på in	1	1
Vet ikke							1	1
		Minske blodtilførsel til		At medikar	1	Iskemi dist	1	1
uten stor b	Presse på a	Stoppe stør	Slippe opp	Vet ikke?	3		1	1
					3		1	1
en blødning	Pas må ligg	Stoppe blø	Om pas fortsetter å bl		3		1	1
ken har kommet ut		Stenge av blødning gjennom å sto			3		1	1
Under føds	Finn lyskep	Mulighet ti	Dersom ma	Har ingen a	1	Hvis en utf	1	1
					3		1	1
					3		1	1
							1	1
					3		1	1
Blødning d	Knyttneve	Stanse blød	Om blødning avtar. Gr		3		1	1
ng etter		Stoppe blo	Se om blød	Smertelind	3		1	1
		Blødningskontroll			1		1	1
Hvis det fo	Legge press	Redde liv. S	Det stoppe	Usikker	1		1	1
		Stoppe blødning			3		1	1
ukontrollert	Hardt press	Klemme av	Minsker bl	Bevissthet	1		1	1
Usikker	Usikker	For og hind	Blødning st	Eventuelt h	3		1	1
Når barn ik	Ca 3cm ove	Redusere/s	Se blødningen reduseres				1	1
Ved nesebl	Knyttneve	Stenge blod	Lyskepuls	Blodtrykksf	3	Blodtrykksf	1	1
?		Stanse livst	?		3		1	1
							1	1
							1	1
ende blødni	Knyttneve/	Avta blødn	Ser at blødningen avta		1	Sirkulasjon	1	1
dette sp er	knyttneve t	stoppe blød	ser at blød	passe på Al	3		1	1
	Ta lyskepul	Hensikten	Blødningen skal ha blitt mindre. Det er vanske				1	1
tter fødsel	Rundt nave	Stoppe blø	Sjekk puls f	Blødningsfa	1	Ingen sirku	1	1
stor blødnin	En hånd/ne	Stoppe blø	Blødninger roer seg.		3		1	1
ukjent	knyttet nev	hindre bloc	sjekke ette	usikker	2		1	1
Når massa	lokalisere l	Begrense b	Se at blødn	Vet ikke	2		1	1
Mindre blø	Knyttneve	Se over	Om blødning avtar, sje		2	Ingen man	1	1

var27	var28	var29	var30	var31	var32	var33	var34	var35
1	2	2	3	1	1	8	1	1
3	1	3	3	4	1	8	1	1
2	2	2	3	1	4	4	5	5
2	2	2	3	2	2	7	3	3
2	2	2	2		5	7	4	6
2	2	2	2		3	5	3	3
2	2	2	2	1	3	6	2	3
2	2	2	2		1			
2	2	2	2	1	8	8	8	8
2	2	2	2	1	8	8	8	8
2	2	2	2	1	1	8	1	1
2	2	2	2					
2	2	2	2	1	6	4	7	6
2	2	2	2	1	2	7	3	3
2	2	2	1	1	3	6	5	4
2	2	2	2	2	4	5	5	4
2	2	2	2	4	3	6	5	4
2	2	2	2	1	2	8	5	5
2	2	2	2	2	3	6		
2	2	2	2	1	2	6	5	5
2	2	2	2	1	5	6	6	5
2	2	2	2	1	7	1	8	8
2	2	2	2		8	8	8	8
2	2	2	2	1	2	2	2	2
2	2	2	2		8	8	8	8
2	2	2	2		8	8	8	8
2	2	2	2		3	6	3	4
2	2	2	2		1	8		
1	3	1	1	3	2	7	2	2
2	2	2	2	3	3	6	3	4
2	2	2	2	1	3	6	5	5
2	2	2	3	1	4	5	4	4
2	2	2	2		1	7	1	1
2	2	2	2	2	1	8	3	3
2	2	2	2	1	1	8	3	2
2	2	3	3	1				
1	1	1	1	4	2	8	2	2
1	2	2	2	2	5	6	5	6
2	2	2	2					
1	2	2	2		6	6	7	6
2	2	2	2	1	6	5	6	6
2	2	2	2	3	3	5	4	5
2	2	1	3		4	6	5	5
3	2	2	2	1	1	8	3	2
2	2	2	2	3	3	6	5	5

1	2	1	2		5	6	4	4
2	2	2	2		5	8	5	3
2	2	2	2	3	4	8	6	8
2	2	2	2		3	5	4	3
2	2	2	2	3	8	8	8	8
2	2	2	2	2	5	5	6	6
2	2	2	2		1	8	2	3
2	2	2	2		2	7	4	4
2	2	2	2	1	8	8	6	6
1	2	2	2		3	8	2	3
2	2	2	2					
2	2	2	2		5	5	5	5
2	2	2	2		5	5	7	5
2	2	2	2	1	4	6	8	5
2	2	2	2					
2	2		2		3	6	3	3
2	2	2	2					
2	2	2	2	1			8	8
2	2	2	2	1	2	7	2	2
2	2	2	2	3	5	5	8	8
2	2	2	2	1	7	5	6	6
1	2	1	3	4	1	8	3	1
1	2	3	3	3	4	8	5	3
2	2	2	2		2	7	1	1
2	2	2	2	1	5	5	6	6
2	2	2	2	1	2	6	5	2
2	2	2	2	1	3	6	6	3
2	2	2	3		7	2	8	7
2	2	2	2	2	3	5	5	4
2	2	2	1	3	3	6	3	3
2	2	2	2		1	7	4	4
2	2	2	3	1	1	7	8	1
2					4	7	2	3
2	2	2	3					
2	2	2	2	2	3	6	5	5
2	2	2	2					
2	2	2	2	2	1	8	2	2
1	2	2	2		1	8	5	
1	2	2	2	1	3	7	3	
2	2	2	2		3	7	4	3
1	2	2	2		2	7	2	2

var36	var37	var38	var39	var40	var41	var42	var43	var44
				1	3		5	
1	4	3	6	1	1	1	5	6
1	1	1	1	1	1	1	6	1
8	4	5	5	4	4	4	4	4
3	5	6	5	2	2	3	4	5
8	3	7	3	1	1	1	2	2
5	5	5	4	2	4	4	4	4
8	3	3	3	2	2	2	2	3
				1	1	1		
8	8	8	8	3	8	4	8	8
8	8	8	8	2	8	8	8	8
8	1	1	1	1	1	1	1	1
				1				
8	6	6	6	3	6	6	6	6
3	5	7	5	1	1	1	5	3
5	4	6	6	3	3	6	5	5
4	4	6	4	1	2	2	2	2
8	3	5	4	3	3	2	2	3
8	8	8	8	1	4	4	4	4
				1		3	3	
4	5	5	5					
8	6	8	8	1	2	1	2	6
8	8	8	8	2	2	2	5	5
8	8	8	8	1	8	1	8	8
8	2	8	2	1	2	1	2	2
8	8	8	8	1	8	1	4	8
8	8	8	8	1	8	1	8	8
8	3	5	4	2	2	3	3	4
			1	1	1	1		
5	5	5	2	1	2	2	5	5
8	8	8	4	1	1	1	3	3
7	4	7	5	2	2	2	2	5
8	8	6	5	1	2	2	3	3
1	1	1	1	1	1	1	1	1
4	4	4	4	1	1	1	1	1
2	3	4	3	3	3	3	4	4
3	4	4	3	1	2	2	3	3
6	6	7	6	2	4	4	3	6
				1	1	1	1	2
7	7	7	7	2	6	6	6	6
6		6	6	1	1	1		
	5	5	5	2	3	2	3	
4	5	4	4	4	4	4	4	5
2	2	4	4	1	1	2	2	3
8	6	6	6	2	6	4	6	6

4	4	4	4	2	3	2	3	3
5	1							
8	8	8	7	1	6	1	8	2
4	5	5	5	1	2	2	3	4
8	8	8	8	8	8	8	8	8
6	5	6	5	3	5	3	5	5
2	2	4	2	3	2	2	2	4
8	4	7	2	2	3	2	3	3
1	5	6	6	1	1	1	1	1
8	3	2	1	2	2	1	3	5
5	5	6	5	4	4	3	5	5
8	8	8	5	4	8	4	8	8
	6	6	6	1	8	3		
				3	3	3	3	6
4	3	6	5	3	3	3	4	3
8	8	8	8	3	8	1	8	6
2	2	2	2	2	2	2	2	2
8	8	8	8	8	8	8	5	5
8	8	8	8	1	3	3	5	5
1	1	3	2	1	1	1	2	2
2	2	7	1	1	3	3	2	4
1				1	1	1	1	1
8	6	6	6	2	6	3	6	5
8	7	7	4	2	1	1	8	5
3	5	7	6	2	2	3	4	3
8	8	8	8	1	8	1	5	6
5	5	6	5	5	3	5	5	5
3	4	3	3	4	4	4	4	4
4	4	5	6	2	3	2	4	6
8	8	8	8	1	1	1	2	2
	5	2	3	1	1	1	1	2
				3				
5	4	5	5	4	5	3	3	6
				1				
2	2	2	2	1	1	1	1	1
5	3	7	5	2	2	1	4	3
3	4		6	5	5	5	8	8
8	5	5	3	2	2	2	4	3
2	3	3	3	2	2	2	3	3

var45	var46	var47	var48	var49	var50	var51	var52	svartid
5								56 sekunde
1	5	2	2	1	1	5	1	23 minutte
1	1	1		1	1	1	1	17 minutte
4	4	4	4	4	4	4	4	1 time 9 mi
5	2	3	3	3	3	3	3	21 minutte
2	1	2	2	2	2	2	2	13 minutte
4	4	3	3	4	4	4	4	19 minutte
2	3	2	2	2	2	2	2	27 minutte
								41 minutte
8	5	8	5	8	1	8	5	29 minutte
8	5	8	5	8	2	4	4	16 minutte
1	1	1	1	1	1	1	1	5 timer 55
								21 minutte
3	3	6	6	3	3	6	4	25 minutte
1	1	5	2	5	1	5	1	21 minutte
5	3	4	4	4	4	4	4	38 minutte
2	2	3	3	3	2	3	3	17 minutte
3	3	2	2	3	3	3	4	16 minutte
7	2	7	7	7	7	7	7	11 minutte
3	4							11 minutte
								4 minutter
1	1	1	1	1	1	1	1	6 minutter
6	3	4	4	4	3	4	4	21 minutte
1	1	8	8	2	8	8	8	17 minutte
2	2	2	2	2	2	2	2	31 minutte
1	1	8	8	1	8	8	8	14 minutte
1	1	8	8	1	8	8	8	13 minutte
5	3	4		2	3	3	3	33 minutte
	1							17 minutte
6	5	5	5	4	4	5	2	28 minutte
1	2	2	1	2	1	2	1	49 minutte
4	4	4	3	3	2	2	2	27 minutte
3	2	3	3	3	2	3	3	24 minutte
1	1	1	1	1	1	1	1	43 minutte
1	1	1	1	1	1	1	1	42 minutte
4	4	4	4	4	4	4	4	58 minutte
								15 minutte
3	2	3	2	2	2	2	2	43 minutte
3	3	5	3	3	3	3	3	1 time 48 s
1	1	1	1	1	1	1	1	1 time 24 n
6	6	7	7	7	7	7	7	7 minutter
								5 minutter
3	3	3	3	3	3	4	4	34 minutte
4	4	4	5	4	4	4	4	22 minutte
2	2	2	2	2	2	2	2	34 minutte
5	4	6	5	5	4	5	4	22 minutte

3	3	3	3	3	3	3	2	48 minutte
								10 minutte
7	2	7	2	2	2	7	2	16 minutte
4	2	5	4	4	3	4	4	31 minutte
8	8	8	8	8	8	8	8	6 minutter
4	4	5	5	3	4	4	4	7 minutter
1	2	2	2	2	2	2	2	25 minutte
2	3	3	2	2	2	3	2	22 minutte
1	1	1	1	1	1	1	1	11 minutte
2	1	2	2	1	1	2	1	10 minutte
								20 minutte
4	4	5	4	4	4	4	4	17 minutte
5	5	7	7	5	6	6	6	50 minutte
								29 minutte
								11 minutte
3	3	4	3	4	4	3	3	16 minutte
								7 timer 12 [illegible]
8	8	8	8	5	8	5	8	46 minutte
2	2	2	2	2	2	2	2	17 minutte
6	6	6	6	6	6	6	6	5 minutter
5	5	5	4	5	4	4	4	28 minutte
2	1	2	2	2	2	2	2	8 minutter
4	5	4	4	4	4	4	4	11 minutte
1	1	2	1	1	1	1	1	8 minutter
4	5	5	5	6	5	6	5	49 minutte
2	2	3	4	2	2	4	5	4 minutter
2	2	3	2	2	2	3	3	8 minutter
2	3	6	6	2	3	6	6	6 minutter
5	5	5	5	5	5	5	5	51 minutte
4	3	3	2	2	3	3	3	55 minutte
4	5	5	5	4	5	5	4	13 minutte
2	2	1	1	1	1	1	1	7 minutter
1	1	1	1	1	1	1	1	7 minutter
								18 minutte
4	4	6	4	5	5	5	5	21 minutte
								17 minutte
1	1	1	1	1	1	1	1	11 minutte
3	3	3	3	3	3	4	4	19 minutte
8	4	4	6	4	4	4	4	3 timer 4 m
3	2	3	3	2	3	2	2	14 minutte
3	3	2	2	3	2	2	2	40 minutte

r 20 sekunder
r 50 sekunder
inutter 49 sekunder
r 33 sekunder

r 28 sekunder
r 29 sekunder
r 10 sekunder
r 28 sekunder
r 56 sekunder
minutter 46 sekunder
r 25 sekunder
r 30 sekunder
r 41 sekunder
r 58 sekunder
r 44 sekunder
r 57 sekunder
r 11 sekunder
r 2 sekunder
52 sekunder
13 sekunder
r 38 sekunder
r 20 sekunder
r 53 sekunder
r 32 sekunder
r 34 sekunder
r 9 sekunder
r 33 sekunder
r 22 sekunder
r 32 sekunder
r 4 sekunder
r 57 sekunder
r 49 sekunder
r 7 sekunder
r 21 sekunder
r 39 sekunder
r 30 sekunder

ninutter 5 sekunder

21 sekunder
r 19 sekunder
r 16 sekunder
r 25 sekunder
r 34 sekunder

r 42 sekunder
r 25 sekunder
r 51 sekunder
r 58 sekunder
25 sekunder
18 sekunder
r 40 sekunder
r 37 sekunder
r 19 sekunder
r 4 sekunder
r 59 sekunder
r 47 sekunder
r 28 sekunder
r 51 sekunder
r 49 sekunder
r 21 sekunder
minutter 51 sekunder
r 30 sekunder
r 9 sekunder
16 sekunder
r 21 sekunder
53 sekunder
r 18 sekunder
41 sekunder
r 24 sekunder
48 sekunder

51 sekunder
r 2 sekunder
r 51 sekunder
r 31 sekunder
12 sekunder

r 22 sekunder

r 29 sekunder
r 29 sekunder
r 40 sekunder
ninutter 5 sekunder
r 18 sekunder
r 47 sekunder
